# Controlling Joint Instability Reduces Inflammatory Pain in Early Knee Osteoarthritis

**DOI:** 10.7759/cureus.87711

**Published:** 2025-07-11

**Authors:** Aya Kuroo, Kenji Murata, Yuri Morishita, Katsuya Onitsuka, Yuichiro Oka, Ken-ichi Tanaka, Naohiko Kanemura

**Affiliations:** 1 Department of Rehabilitation, University of Human Arts and Sciences, Saitama, JPN; 2 Department of Physical Therapy, Saitama Prefectural University, Koshigaya, JPN; 3 Department of Rehabilitation, Tokyo Kasei University, Sayama, JPN; 4 Department of Physical Therapy, Tohto University, Chiba, JPN; 5 Department of Rehabilitation Science, Hokkaido University, Sapporo, JPN; 6 Department of Health and Social Services, Saitama Prefectural University, Koshigaya, JPN

**Keywords:** calcitonin gene-related peptide, controlled abnormal movement, dorsal root ganglion, early knee oa, inflammatory pain, joint instability, neuropathic pain, osteoarthritis, pain

## Abstract

Background and aim

Knee osteoarthritis (OA) is characterized by joint deformity and pain, both of which exert physical and psychological effects on affected individuals. The pain that often appears in the early stages is influenced by the production of inflammatory pain-related factors that respond to nociceptive stimulation from the periarticular tissues. These factors are expressed in small cells within the dorsal root ganglion (DRG) of the spinal cord. Furthermore, neuropathic pain involved in knee OA pain is influenced by neuropathic pain-related factors expressed in DRG small and medium cells, as well as inflammatory pain-related factors expressed in medium cells, which induce chronic pain. However, how these pain-related factors change during the progression of knee OA remains unclear. In addition, joint instability accelerates the progression of knee OA. Reduction of instability reduces mechanical stimulation and delays cartilage degeneration. However, the effect of pain suppression is unknown and requires further investigation. This study aimed to clarify some of the pathological changes in pain generation in the knee OA stage and verify the effect of joint instability suppression on pain reduction.

Materials and methods

Forty-seven adult Wistar rats were divided into OA, controlled abnormal joint movement (CAM), and sham groups, then tissues were collected at four and 12 weeks postoperatively. Knee OA was induced by anterior cruciate ligament transection. In CAM, a tibial suture controlled anterior displacement post-transection. Joint instability was assessed using soft radiography. Histological analysis of the knee joints, fluorescent immunohistochemistry of DRG for inflammatory and neuropathic pain-related factors, and assessments of pain behavior were performed. One-way analysis of variance (ANOVA) followed by Tukey’s multiple comparison test was used to evaluate joint instability and DRG-positive cells-related pain. The Kruskal-Wallis test with Steel-Dwass multiple comparisons was used for histological data. Pain behavior was evaluated using a mixed-design two-way ANOVA.

Results

Knee joint instability was greatest in the OA compared with the CAM and sham. Articular cartilage degeneration was significantly more severe in the OA and CAM than in the sham at both time points. Calcitonin gene-related peptide (CGRP) expression in the DRG was higher in the OA than in the sham at four weeks postoperatively. No significant differences were observed in substance P, isolectin B4, and P2X3 expressions across groups or time points. Pain-related factors were mostly expressed in the small DRG cells. The paw withdrawal thresholds for pain behavior decreased immediately postoperatively, improved by two weeks, and decreased significantly again at 12 weeks postoperatively; however, no significant differences between the groups were noted.

Conclusion

CGRP expression in early knee OA contributes to inflammatory pain, and reacquisition of joint stability suppresses inflammatory pain. In this study, neuropathic pain, including allodynia, was not observed in advanced knee OA.

## Introduction

Knee osteoarthritis (OA) is one of the most common joint diseases, characterized by joint deformity and pain with both physical and psychological influences [1,2]. Among affected individuals, approximately 25% of men and 33% of women experience pain [3]. Pain often arises in the early stages of knee OA, becomes persistent and chronic, and is a significant factor limiting activity. However, effective treatments for knee OA pain have not yet been established and represent an unmet medical need [4,5].

Knee OA pain is caused by nociceptive stimulation from the knee joint. When inflammation occurs, the healing process causes tissue fibrosis with increased vascularity, accompanied by an increase in the number of nerve fibers with free nerve endings, resulting in pain. Additionally, age-related fibrosis of the surrounding tissues lowers the pain threshold, causing pain even in the absence of inflammation. While some joint lesions, such as periostitis and bone marrow lesions, contribute to pain [6,7], the relationship between structural changes in knee OA and pain remains unclear [6-9].

In knee OA, cytokines released by inflammatory stimuli cause hyperalgesia and stimulate chondrolytic protease production to degrade the extracellular matrix [10]. Nociceptor stimulation excites C fibers in the sensory nerves, which transmit pain signals to the dorsal root ganglia (DRG) of the spinal cord. Unmyelinated C fibers extend from small cells in the DRG and produce neuropeptides, such as calcitonin gene-related peptide (CGRP) and substance P (SP). Expression of CGRP and SP in small DRG cells indicates inflammatory pain.

Medium- to large-sized cells in the DRG produce Aβ fibers and transmit tactile and pressure sensations via mechanoreceptors. When a nociceptive stimulus excites Aβ fibers, the tactile sensation is perceived as pain. This is one of the causes of allodynia, in which pain is felt even when the stimulus is not painful, as detected by the von Frey test [11]. CGRP is also expressed in DRG medium cells in a rat knee OA model and is thought to transmit nociceptive stimuli through nerve fibers different from those of small cells [12]. Additionally, small cells with non-peptide-dependent nociceptors are targeted by isolectin B4 (IB4) and are involved in chronic pain [13]. Furthermore, IB4-positive cells co-express the purine receptor P2X3, which is associated with neuropathic pain. Neuropathic pain is also linked to chronic pain [14].

Cartilage damage is caused by joint instability [2], and the progression of cartilage degeneration depends on the severity of joint instability [15]. Anterior withdrawal of the tibia is one of the indicators of joint instability in the sagittal plane. A previous study has shown that cartilage degenerates four weeks after anterior cruciate ligament (ACL) transection, resulting in early-stage OA with low Osteoarthritis Research Society International (OARSI) scores, and joint deformity progresses to moderate cartilage matrix damage and chondrocyte loss at 12 weeks postoperatively, resulting in advanced-stage OA [16]. In addition, the aforementioned study reported that reacquisition of joint stability in the sagittal plane delays cartilage degeneration in knee OA.

Conversely, the effect of reacquiring joint stability on pain has not been considered, and its effectiveness needs to be verified. Additionally, increased CGRP expression was found in the L4 DRG, where intra-articular innervated neuronal cell bodies are located in early OA; however, changes in the factors causing pain according to the stage of knee OA remain unclear [17].

In this study, we aimed to clarify some of the pathological changes involved in pain generation in the knee OA stage and verify the pain-suppressing effect of joint instability control. We hypothesized that early-stage knee OA would show increased expression of inflammatory pain-related factors in the dominant nerve area within the joint space and that advanced-stage knee OA would show increased expression of both inflammatory and neuropathic pain-related factors in DRG medium cells. Furthermore, the reacquisition of joint stability may suppress mechanical stimuli and inflammation associated with cartilage degeneration, thereby reducing inflammatory pain.

## Materials and methods

Animals and experimental design

**Figure 1:**
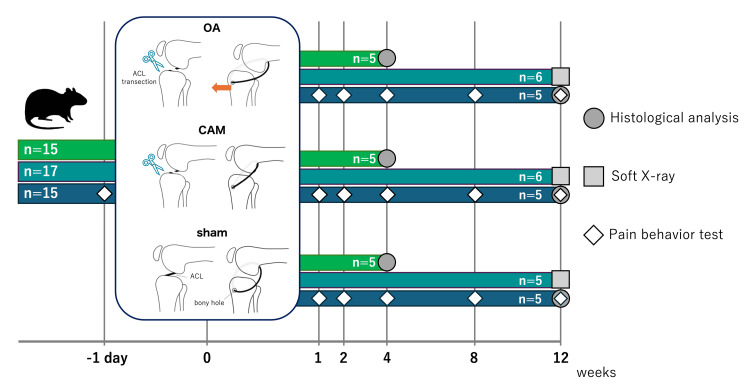
Experimental protocol. Eleven-week-old male Wistar rats were divided into the following three groups: knee osteoarthritis (OA) (n=16), controlled abnormal joint movement (CAM) (n=16), and sham (n=15) groups. Tissue samples from 15 rats were collected at four weeks postoperatively (n=5 for each group), with the remaining samples collected at 12 weeks postoperatively. Seventeen rats designated for 12-week tissue collection underwent radiography (OA=6, CAM=6, sham=5), and pain-related behaviors were evaluated in 15 rats (n=5 for each group). OA: osteoarthritis, CAM: controlled abnormal joint movement

This study was conducted in accordance with the Animal Research: Reporting In Vivo Experiments (ARRIVE) guidelines and the Institutional Guidelines and was approved by the Animal Research Ethics Committee of Saitama Prefectural University (approval number: 28-2). A total of 47 11-week-old male Wistar rats (Shizuoka, Japan: Japan SLC Inc.) were randomly divided into the following three groups: knee OA (n=16), controlled abnormal joint movement (CAM) (n=16), and sham (n=15) groups. The animals were assigned to two postoperative time points - four and 12 weeks - for tissue collection (n=15 per group), and an additional 15 rats were evaluated for pain-related behaviors. Rats were allowed full cage activity, and the room temperature was maintained at 23±2°C with a 12-h light-dark cycle. The study design is shown in Figure 1.

Surgical induction

Anesthesia was first performed by induction anesthesia with isoflurane. Then, the rats were injected subcutaneously with an anesthetic mixture of medetomidine (0.375 mg/kg), midazolam (2.0 mg/kg), and butorphanol (2.5 mg/kg). As described previously, knee OA was induced by transecting the anterior cruciate ligament (ACL) [16,18]. CAM was performed by cutting the ACL and inserting a nylon suture 3-0 (Tokyo, Japan: Akiyama Corporation) through a bone hole in the tibia to control the anterior withdrawal of the tibia. In the sham model, the joint capsule was cut, and a bone hole was formed in the tibia. The right hind limb was used as the target limb in all animals.

Joint instability assessment

Tissue samples were collected 12 weeks after surgery. Euthanasia was carried out through the administration of an overdose of isoflurane. At 12 weeks postoperatively, a soft X-ray (M-60; Tokyo, Japan: Softex Co.) was used to measure anterior tibial translation as joint instability. Sagittal plane stress imaging was performed using a lower leg detached just below the greater trochanter of the femur, with the knee joint fixed at 90° flexion and the proximal tibia pulled forward using a 0.2 kgf calibration spring. Soft X-rays were applied at 28 kV and 1 mA for 1 s, and the amount of anterior tibial translation was measured from the images using Image J (Bethesda, MD: National Institutes of Health).

Histological analysis

The right knee joint and spine (L1-L6) were fixed in 4% paraformaldehyde phosphate buffer for 48 h and one week, respectively. The right knee joint was demineralized using 10% ethylenediaminetetraacetic acid, while the spine was demineralized using Super Decalcifier I/Delicate (Warrington, PA: Polyscience). After scroll replacement, each tissue was embedded in Tissue-Tek O.C.T. Compound (Tokyo, Japan: Sakura Finetek Japan). Frozen consecutive sections of the right medial knee joint on the sagittal plane and the spine on the horizontal plane, including the DRG of the spinal cord, were prepared at a thickness of 14 μm using Cryostat CM3050S (Wetzlar, Germany: Leica).

The knee joint was histologically evaluated using the OARSI score following Safranin O-Fast Green staining [19]. Cartilage and subchondral bone damage grades were assessed by two evaluators blinded to the stained images.

Immunofluorescence staining was performed to observe the expression of pain-related factors in the DRGs. The primary antibodies used were rabbit anti-SP polyclonal antibody (1:100; Woburn, MA: BIOSS), mouse anti-CGRP monoclonal antibody (1:1000; Cambridge, UK: Abcam plc), rabbit anti-P2X3 polyclonal antibody (1:1000; Cambridge, UK: Abcam plc), and IB4 (Griffonia simplicifolia Lectin I-B4 Isolectin, FITC Conjugate) (1:200; Newark, CA: Vector Laboratories). The secondary antibodies were goat-derived anti-rabbit IgG DyLight 488 (1:500; Yokohama, Japan: Thermo Fisher Scientific), goat-derived anti-mouse IgG Alexa Fluor 594 (1:1000; Tokyo, Japan: Life Technologies), and goat-derived anti-rabbit IgG Alexa Fluor 546 (1:1000; Tokyo, Japan: Life Technologies). Double staining was performed in pairs of SP and CGRP, IB4 and P2X3. The number of positive cells per DRG area was determined using an all-in-one fluorescence microscope BZ-X700 (Osaka, Japan: KEYENCE). Positive cells were classified by size into small (<500 μm^2^), medium (500-950 μm^2^), and large (≥950 μm^2^) categories, based on previous studies, and the percentage of each size category among the positive cells was calculated [20].

Pain behavior test

The von Frey test was performed preoperatively (baseline) and at one, two, four, eight, and 12 weeks postoperatively. All rats were acclimated to the environment for 30 min prior to testing. The filaments were then stimulated for 5 s. During this time, we determined whether the rats exhibited nociceptive behaviors, such as lifting or biting the hind paw. The 50% paw withdrawal threshold (PWT) was determined based on a previous study [21].

Statistical analysis

All statistical analyses were performed using R version 4.4.2 (Vienna, Austria: The R Foundation for Statistical Computing). Joint instability was evaluated using a one-way analysis of variance (ANOVA) followed by multiple comparisons using the Tukey method. Histological analysis of the knee joints was performed using the Kruskal-Wallis test, with the Steel-Dwass test applied for multiple comparisons. One-way ANOVA was used to analyze the number of positive cells in the DRGs, and post-hoc testing was conducted using the Tukey method. For pain behavior, outliers were identified based on the baseline measurements of the von Frey test, and data from rats containing outliers were excluded. Outliers were identified using the interquartile range (IQR) method, defined as values below the first quartile minus 1.5 times the IQR or above the third quartile plus 1.5 times the IQR. This approach was chosen because of the non-normal distribution of the data. The remaining data were normalized to baseline values, and a mixed-design two-way ANOVA was performed. The significance level α for all statistical tests is 0.05.

## Results

Joint instability

**Figure 2:**
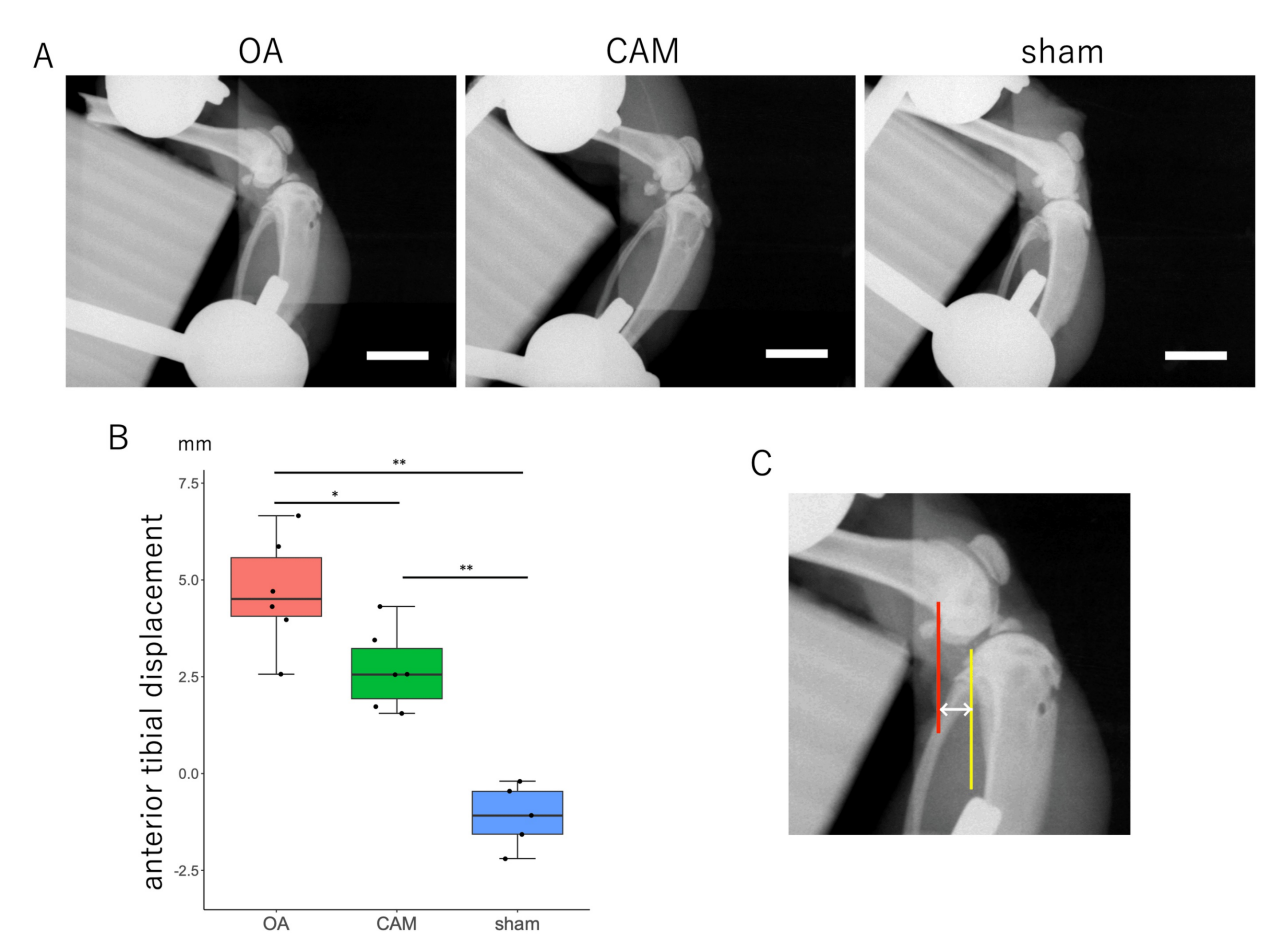
Joint instability as anterior tibial translation. *P<0.05 OA group vs. CAM group. **P<0.01 OA group vs. sham group, CAM group vs. sham group. (A) Radiographic image of tibial anterior translation (scale bar=10 mm). (B) Joint instability in the sagittal plane. The OA group exhibited the greatest joint instability among the three groups, while the CAM group showed reacquisition of joint stability. (C) Measurement of anterior tibial translation: the distance from the posterior aspect of the lateral femoral condyle (red line) to the posterior aspect of the lateral tibial condyle (yellow line). OA: osteoarthritis; CAM: controlled abnormal joint movement

At 12 weeks postoperatively, the OA and CAM groups exhibited greater anterior tibial translation than the sham group, and the OA group exhibited the greatest instability among the three groups. This indicated joint stability reacquisition (F {2, 14} = 35.02, p<0.001; OA vs. CAM, p<0.05; OA vs. sham, p<0.001; CAM vs. sham, p<0.001) (Figures 2A-2C). Anterior tibial translation was measured as the distance the posterior aspect of the lateral tibial condyle moved relative to the posterior aspect of the lateral femoral condyle (Figure 2C).

Cartilage degeneration and subchondral bone injury

The intraclass correlation coefficient between the two examiners who requested OARSI scoring was intraclass correlation coefficient (ICC) (2,1)=0.81. A comparison of OARSI scores for cartilage and subchondral bone injuries is presented in Table 1. Degeneration of both cartilage and subchondral bone was more prominent in the tibia than in the femur.

**Table 1:** OARSI score of cartilage and subchondral bone. OA: osteoarthritis; CAM: controlled abnormal joint movement; OARSI: Osteoarthritis Research Society International

Variables		Kruskal-Wallis χ^2^	df	p-Value	Median		Steel-Dwass p-Value		r-Value
Cartilage	4 weeks	10.316	2	0.0058	OA	8.67	OA-CAM	0.4607	0.46
					CAM	3.67	OA-sham	0.0014	0.99
					sham	0.33	CAM-sham	0.0024	0.98
	12 weeks	11.281	2	0.0036	OA	8.33	OA-CAM	0.1161	0.73
					CAM	3.33	OA-sham	0.0048	0.97
					sham	0.67	CAM-sham	0.0015	0.97
Subchondral bone	4 weeks	3.8168	2	0.1483	OA	1.33	OA-CAM	0.6609	0.35
					CAM	1.00	OA-sham	0.0925	0.74
					sham	0.00	CAM-sham	0.7345	0.29
	12 weeks	9.2149	2	0.01	OA	6.00	OA-CAM	0.0396	0.88
					CAM	0.33	OA-sham	0.0123	0.97
					sham	0.00	CAM-sham	0.8482	0.21

**Figure 3:**
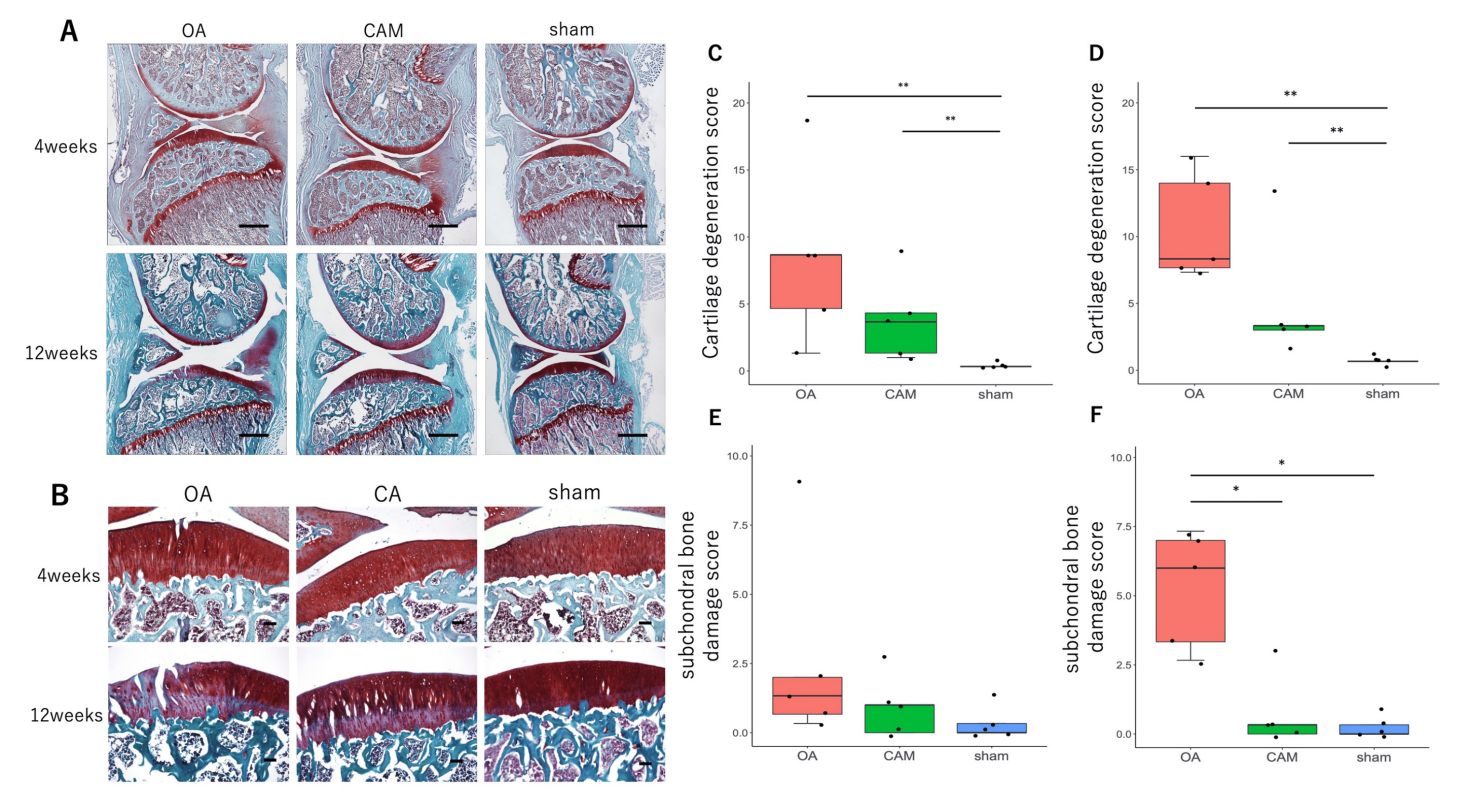
Cartilage degeneration and subchondral bone damage. *P<0.05 subchondral bone injury at 12 weeks, OA group vs. sham group, OA group vs. CAM group. **P<0.01 cartilage damage at four and 12 weeks, OA group vs. sham group, CAM group vs. sham group. (A) Knee sections were stained with Safranin O and Fast Green. The OA group exhibited cartilage degeneration beginning at four weeks, along with subchondral bone damage. At 12 weeks, both cartilage degeneration and subchondral bone damage had progressed. In contrast, no obvious subchondral bone damage was observed in the CAM group up to 12 weeks (scale bar=1 mm). (B) Magnified image of the middle part of the tibial articular surface (scale bar=100 μm). The OARSI scores of cartilage damage at four weeks (C) and 12 weeks (D). Scores were higher in the OA and CAM groups than in the sham group. Subchondral bone OARSI scores at four weeks (E) and 12 weeks (F). The OA group showed significantly greater scores than the other groups at 12 weeks. OA: osteoarthritis; CAM: controlled abnormal joint movement; OARSI: Osteoarthritis Research Society International

Regarding cartilage degeneration, the OA group exhibited cartilage surface irregularity, cracking, or loss at four weeks postoperatively. The CAM group showed slight cartilage surface irregularity at 12 weeks postoperatively, while the sham group maintained a smooth cartilage surface at both time points (Figures 3A, 3B). The OARSI cartilage damage scores were significantly higher in the OA and CAM groups than in the sham group at both four and 12 weeks postoperatively (Figures 3C, 3D). In contrast, no significant differences were observed between the OA and CAM groups at either time point (four weeks χ^2^=10.316, p<0.01; OA vs. CAM, p=0.4607; OA vs. sham, p<0.01; CAM vs. sham, p<0.01; 12 weeks χ^2^=11.281, p<0.01; OA vs. CAM, p=0.1161; OA vs. sham, p<0.01; CAM vs. sham, p<0.01). For subchondral bone injuries, an increase in basophils within the tidemark was observed in the OA group at four weeks postoperatively. Additionally, tidemark division and fibroblasts were present in the bone marrow at 12 weeks postoperatively. No obvious subchondral bone damage was observed in the CAM group until 12 weeks postoperatively, and no degeneration was observed in the sham group (Figure 3B). At four weeks, there were no significant differences in OARSI scores between the groups (χ^2^=3.8168, p=0.1483; OA vs. CAM, p=0.6609; OA vs. sham, p=0.0925; CAM vs. sham, p=0.7345). However, at 12 weeks, the OA group showed significantly higher scores than both the CAM and sham groups (χ^2^=9.2149, p<0.05; OA vs. CAM, p<0.05; OA vs. sham, p<0.05; CAM vs. sham, p=0.8482) (Figures 3E, 3F).

Fluorescence immunohistochemistry

**Figure 4:**
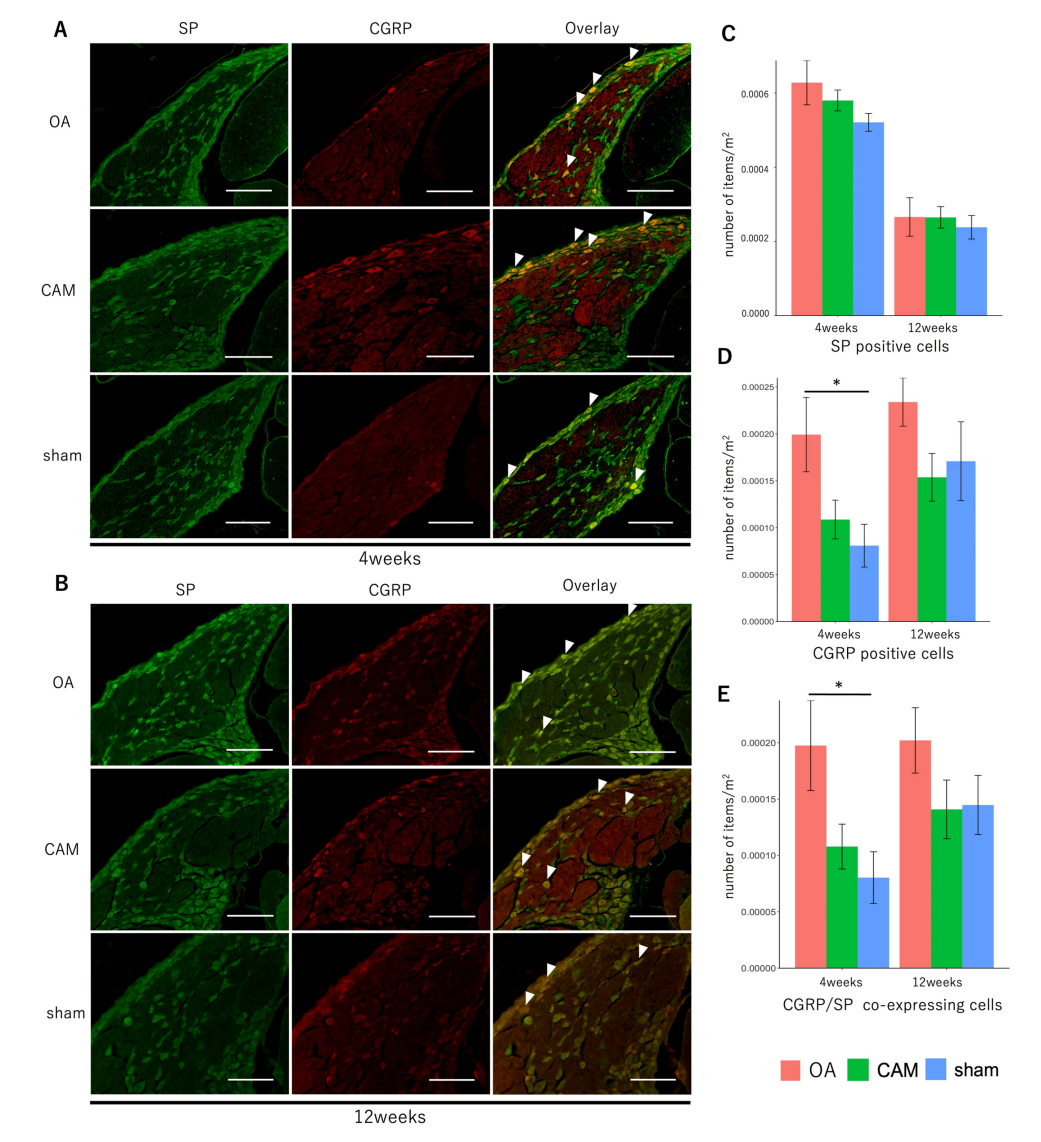
SP- and CGRP-positive cells for pain-related factors in DRG. *P<0.05 OA group vs. sham group in CGRP-positive cells and cells co-expressing CGRP and SP. (A, B) Immunofluorescence staining of SP and CGRP in DRG at four and 12 weeks. Arrowheads indicate coexpressing cells (scale bar=200 μm). (C-E) Number of SP- and CGRP-positive cells and their co-expression. The number of CGRP-positive cells and the coexpression of SP and CGRP at four weeks were higher in the OA group than in the sham group. OA: osteoarthritis; CAM: controlled abnormal joint movement; SP: substance P; CGRP: calcitonin gene-related peptide; DRG: dorsal root ganglion

**Figure 5:**
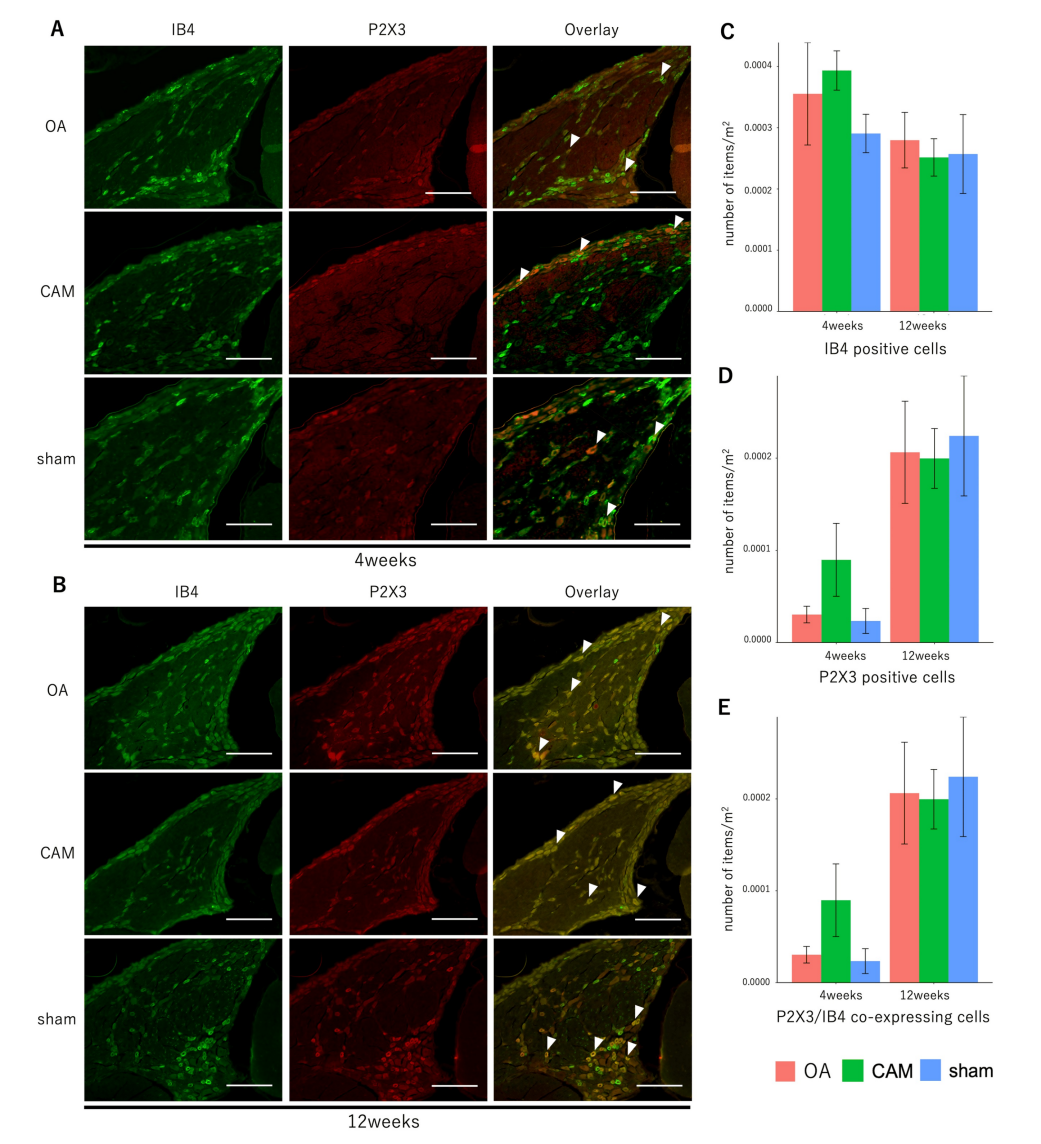
IB4 and P2X3 positive cells for pain-related factors in DRG. (A, B) Immunofluorescence staining of IB4 and P2X3 in DRG at four and 12 weeks. Arrowheads indicate coexpressing cells (scale bar=200 μm). (C-E) Number of IB4 and P2X3 positive cells per unit area in DRG. There were no significant differences in the number of IB4- and P2X3-positive cells. OA: osteoarthritis; CAM: controlled abnormal joint movement; IB4: isolectin B4; CGRP: calcitonin gene-related peptide; DRG: dorsal root ganglion

Staining images and the number of positive cells per unit area for pain-related factors, SP and CGRP, and IB4 and P2X3 in the DRG are shown in Figures 4A-4E, 5A-5E. There was no significant difference in the number of SP-positive cells between the groups at four and 12 weeks postoperatively (four weeks: F {2, 12}=1.7489, p=0.2155; 12 weeks: F {2, 12=0.1629}, p=0.8515). Cell size distributions of SP-positive cells did not differ significantly between groups, with small cells comprising the majority at both time points (four weeks: F {2, 12}=1.1743, p=0.3422; 12 weeks: F {2, 12}=0.2194, p=0.8061). The number of CGRP-positive cells was significantly higher in the OA group than in the sham group at four weeks postoperatively (F {2, 12}=4.5619, p<0.05, OA vs. CAM, p=0.109, OA vs. sham, p<0.05, CAM vs. sham, p=0.7792), while no significant differences were observed at 12 weeks (F {2, 12}=1.7403, p=0.217). The CGRP-positive cell size distribution also showed a predominance of small cells in all groups, both four and 12 weeks postoperatively (four weeks: F {2, 12}=1.5865, p=0.2447; 12 weeks: F {2, 12}=0.2387, p=0.7913). Co-localization of SP and CGRP was more frequently observed in the OA group than in the sham group four weeks postoperatively (F {2, 14}=4.4657, p<0.05; OA vs. CAM, p=0.1141; OA vs. sham, p<0.05; CAM vs. sham, p=0.784). No significant difference was observed among groups at 12 weeks (F {2, 12}=1.5963, p=0.2428).

For IB4 and P2X3, no significant differences were observed among the three groups in the number of positive cells or in their coexpression at either time point. Specifically, for IB4: four weeks F (2, 12)=0.9004, p=0.4322; 12 weeks F (2, 12)=0.0938, p=0.9112. For P2X3: at four weeks F (2, 12)=2.1752, p=0.1563; at 12 weeks F (2, 12)=0.0575, p=0.9444. For coexpression of IB4 and P2X3: at four weeks F (2, 12)=2.7995, p=0.1005; at 12 weeks F (2, 12)=0.0778, p=0.9256.

Pain behavior test

**Figure 6:**
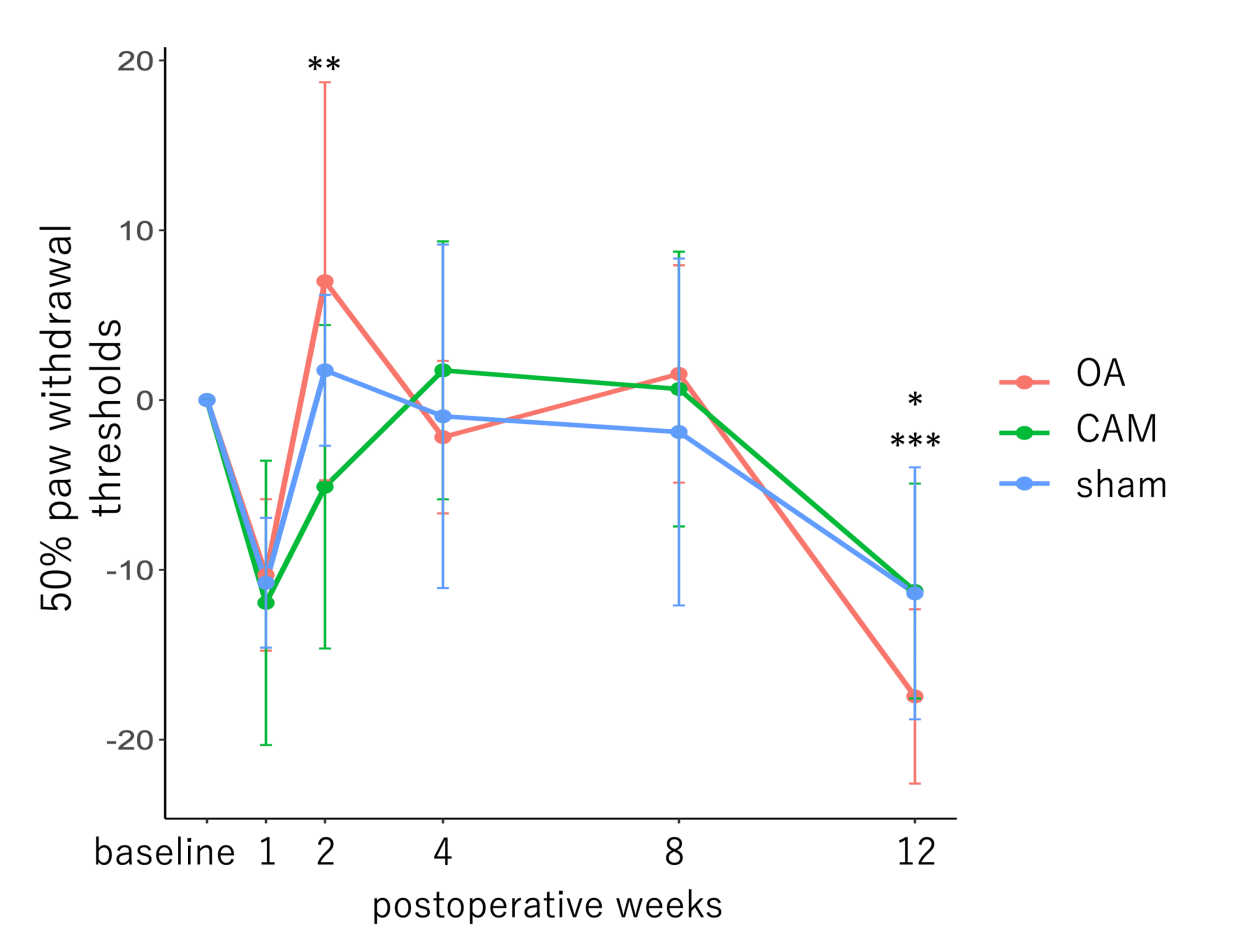
Pain behavior test (von Frey test). *P<0.05 at baseline vs. 12 weeks. **P<0.05 at one vs. two weeks. ***P<0.001 at four vs. 12 weeks. The 50% PWT values were normalized to baseline measurements. Postoperative pain thresholds improved after two weeks; pain thresholds at 12 weeks were lower than those at baseline. OA: osteoarthritis; CAM: controlled abnormal joint movement; PWT: paw withdrawal threshold

After excluding one sham data point due to outlier baseline measurements, the 50% PWT was compared across time points. A significant main effect of postoperative time was observed (F {5, 70}=6.02, p<0.001) (Figure 6). Although the 50% PWT decreased immediately after surgery in all rats, the difference was not statistically significant. The threshold improved at two weeks postoperatively (one week vs. two weeks, p=0.0286; baseline vs. two weeks, p=1.000), but declined again after eight weeks. Pain thresholds were significantly lower at 12 weeks postoperatively (baseline vs. 12 weeks, p<w0.05; four weeks vs. 12 weeks, p<0.001; eight weeks vs. 12 weeks, p<0.001). In contrast, there were no significant main effects between groups (F {2, 14}=0.004, p=0.996) or interaction effects between groups and time points (F {10, 70}=0.529, p=0.864).

## Discussion

Cartilage degeneration was observed from four weeks postoperatively in both the OA and CAM groups, consistent with a previous study reporting that OA begins at four weeks postoperatively in an ACL transection-induced OA model [15]. The increase in OARSI scores in the CAM group was slower compared to that in the OA group up to 12 weeks postoperatively. Although the difference between the two groups was not statistically significant, the slower progression in the CAM group may reflect reduced cartilage degeneration, as reported in previous studies [16]. Subchondral bone damage was observed exclusively in the OA group and was accompanied by high OARSI scores, particularly at 12 weeks postoperatively. The overall severity of cartilage and subchondral bone damage indicated that the four-week postoperative OA and CAM groups, as well as the 12-week postoperative CAM group, represented early-stage OA, while the 12-week postoperative OA group reflected advanced-stage OA.

In knee OA, inflammatory pain predominates in the early stage, while neuropathic pain typically emerges in the advanced stage [22]. Although knee OA pain is often classified and discussed independently, some factors expressed by inflammation, such as CGRP and nerve growth factors, affect neuropathic pain by acting on tactile transmission, nerve activity, and nerve branch sprouting [23]. Because some pain factors coexist and influence each other, it is difficult to clearly distinguish their functions. However, in this study, pain was classified according to the primary function of each factor and the size of the cells that express them. We compared the expression of SP and CGRP, which are inflammatory pain-related factors. SP expression showed no significant difference between the groups at four and 12 weeks postoperatively. Although no statistical comparisons were performed, the incidence of SP at 12 weeks postoperatively was reduced to approximately half of that observed at four weeks in all groups. Considering that the L4 DRG receives more extensive innervation from the joint cavity, the observed changes in SP incidence at four and 12 weeks postoperatively across all groups are likely attributable to joint capsule disruption. These changes may reflect the resolution of inflammation induced by the capsular injury. Conversely, CGRP was significantly more highly expressed in the OA group than in the sham group at four weeks postoperatively, suggesting that inflammatory pain occurred in the OA group. Furthermore, the higher coexpression of SP and CGRP in the OA group strongly supports the involvement of inflammatory pain. In contrast, the CAM group did not significantly differ from the sham group in CGRP expression, indicating that controlling joint instability may prevent inflammatory pain. Additionally, at 12 weeks postoperatively, there was no difference in the number of CGRP-positive cells between the groups, suggesting that inflammatory pain had decreased in the OA group as the disease progressed, while inflammatory pain persisted in the CAM group due to the maintenance of an early disease stage. CGRP is involved in both inflammatory and neuropathic pain [24]; it is expressed in small cells associated with C fibers during inflammatory pain and in medium cells associated with Aβ fibers during neuropathic pain [25]. In this study, positive cells were expressed mainly in small cells, and the proportion of small cells did not differ among the groups, suggesting that CGRP expression may contribute to inflammatory pain.

IB4 targets non-peptide-dependent neurons involved in neuropathic pain and is mostly distributed in small cells [26]. In the present study, there were no significant differences in the number of IB4-positive cells or in the percentage of cell size among groups. These findings are consistent with a previous study that reported no increase in IB4 in OA rats [12]. Although most P2X3-positive cells coexpressed IB4, there were no significant differences in cell number, size, or proportion between the models, which does not support the involvement of IB4 and P2X3 co-localization in neuropathic pain [27].

The 50% PWT measured using the von Frey test suggests that the pain threshold was lowered due to surgical invasion during the early postoperative period. At two weeks postoperatively, the 50% PWT was inferred to have improved because of the quiescence of inflammation caused by surgical invasion. At four weeks postoperatively, OA changes were histologically confirmed; however, intra-articular inflammation associated with OA changes had no effect on the 50% PWT. This test was applied to the plantar surface and may have been inadequate to reflect inflammatory pain, which is more likely to respond locally (in the knee joint). Similarly, histological OA progression was observed in the OA group at 12 weeks postoperatively; however, the threshold decreased in all groups, suggesting that OA progression was not directly responsible for the decrease in 50% PWT. It is also possible that hypersensitivity to von Frey hair stimulation is responsible. In fact, it has been reported that prolonged von Frey testing increases hypersensitivity to stimuli [21]. The high expression of P2X3 in the DRG at 12 weeks postoperatively in all groups may have reflected this. A decrease in 50% PWT can be induced not only by inflammatory pain but also by allodynia, and the threshold is considered to be ≤4 g [21]. Allodynia is generally caused by central sensitization in the dorsal horn of the spinal cord and is associated with neuropathic pain [28]. In the present study, some rats in the OA group exhibited a 50% PWT of ≤4 g at 12 weeks postoperatively; however, the baseline-normalized values showed no significant differences between groups and were not sufficient to indicate the presence of allodynia. The fact that the expression of pain-related factors in DRG medium cells did not differ significantly among the three groups also supports the absence of allodynia.

This study has three limitations. The first is a problem related to the innervating nerves. The effect of nerves innervating the outside of the knee capsule in the L4DRG cannot be ignored, as the intra-articular innervating nerves were not identified. Additionally, we only analyzed the L4DRG, which is considered to have many nerves innervating the knee joint cavity, and we were unable to analyze pain originating outside the joint capsule [29]. Second, the effect of central sensitization was not adequately verified; it is difficult to detect central sensitization other than allodynia with the von Frey test. The dynamics of molecular biological parameters higher up the spinal cord need to be investigated. Third, the sample size was small due to high variability in some measurements, which may have prevented a full examination of the effects of the intervention.

In the future, it will be necessary to expand the scope of the analysis to include subchondral bone innervations that are thought to be associated with knee OA pain. Furthermore, the impact of neuropathic pain in knee OA should be examined, including analysis of molecular parameters involved in central sensitization in the dorsal horn of the spinal cord.

## Conclusions

In the knee OA induced by ACL transection, early-stage OA occurred at four weeks postoperatively. In DRG, the number of CGRP-positive cells, the distribution of small cells among positive cells, and the coexpression of CGRP and SP were highest in the OA group, indicating that OA causes early inflammatory pain. In CAM, CGRP expression was not significantly different but was lower in the OA group. At 12 weeks postoperatively, there were no significant group differences in the expression of inflammatory and neuropathic pain-related factors in the DRG. Furthermore, CAM showed a slow progression of OA. In all groups, 50% PWT ruled out the occurrence of allodynia. In conclusion, the reacquisition of joint stability is expected to reduce inflammatory pain in early OA.
